# Is genetic fatherhood within reach for all azoospermic Klinefelter men?

**DOI:** 10.1371/journal.pone.0200300

**Published:** 2018-07-25

**Authors:** Veerle Vloeberghs, Greta Verheyen, Samuel Santos-Ribeiro, Catherine Staessen, Willem Verpoest, Inge Gies, Herman Tournaye

**Affiliations:** 1 Centre for Reproductive Medicine, Universitair Ziekenhuis Brussel, Vrije Universiteit Brussel, Brussels, Belgium; 2 Centre for Medical Genetics, Universitair Ziekenhuis Brussel, Vrije Universiteit Brussel, Brussels, Belgium; 3 Department of Pediatrics, Universitair Ziekenhuis Brussel, Vrije Universiteit Brussel, Brussels, Belgium; University Hospital of Münster, GERMANY

## Abstract

**Background:**

Multidisciplinary management of Klinefelter cases is now considered good clinical practice in order to ensure optimal quality of life. Reproductive performance of Klinefelter men is an important issue however literature in this domain is limited and prone to bias.

**Study design:**

This was a retrospective longitudinal cohort study performed at a tertiary referral University Centre for Reproductive Medicine and Genetics. One hundred thirty-eight non-mosaic azoospermic Klinefelter patients undergoing their first testicular biopsy (TESE) between 1994 and 2013, followed by intracytoplasmic sperm injection (ICSI) with fresh or frozen-thawed testicular sperm in the female partner, were followed-up longitudinally. The main outcome measure was cumulative live birth rate per Klinefelter patient embarking on TESE-ICSI.

**Findings:**

In forty-eight men (48/138) sperm were successfully retrieved at the first TESE (34.8%). The mean age of the patients was 32.4 years. Younger age at first TESE was associated with a higher sperm retrieval rate (p<0.001). Overall 39 couples underwent 62 ICSI cycles and 13 frozen embryo transfer cycles resulting in in 20 pregnancies and 14 live birth deliveries (16 children). The mean age of the female partner was 28.1 years. The crude cumulative delivery rate after four ICSI cycles was 35.9%. Per intention-to-treat however, only 10.1% (14/138) of the Klinefelter men starting treatment succeeded in having their biologically own child(ren).

**Conclusion:**

Non-mosaic Klinefelter patients with azoospermia seeking treatment by TESE-ICSI should be counseled that by intention-to-treat the chance of retrieving sperm is fair, however only a minority will eventually father genetically own children.

## Introduction

Today care of men with Klinefelter Syndrome (KS) has become a multidisciplinary approach involving endocrinologists, paediatricians, psychologists, urologists as well as reproductive specialists [1]. KS is one of the most prevalent genetic causes of infertility (11%) in azoospermic males and accounts for 1 to 2% of infertility in the overall male population [2,3]. Many KS patients are diagnosed only at adult age due to their fertility problem [4]. In general KS patients are azoospermic as a result of progressive fibrosis and hyalinization of seminiferous tubules and concomitant loss of spermatogonial stem cells [5,6]. Only few have retained spermatogenic ability compatible with the presence of spermatozoa in the ejaculate [7]. A recent study of Rohayem et al. did not find any difference in a number of clinical and biochemical parameters in KS patients positive or negative for sperm in the ejaculate [8].

Nevertheless KS men may have residual foci with preserved spermatogenesis in their testis and by performing a testicular sperm extraction (TESE) occasional tubules showing active spermatogenesis may be recovered [9].

The introduction of intracytoplasmic sperm injection (ICSI) with testicular sperm in azoospermic men with KS, allowed fatherhood in these men previously considered sterile [9] and this treatment strategy has become a routine procedure at many fertility centres. A recent meta-analysis reported a testicular sperm retrieval rate of 43% (39% - 48%) in KS men with no differences observed between conventional testicular sperm extraction (cTESE) or microsurgical TESE (micro-TESE) [10].

Although TESE-ICSI in KS patients has been introduced more than two decades ago [9] the methodological issue of dichotomy bias is encountered in most studies focusing on reproductive outcome in these patients. The data in literature is limited either to sperm retrieval rates following consecutive TESE procedures or to outcome of ICSI exclusively in selected good prognosis KS men. In both of these situations sufficient testicular sperm is obtained, biasing both reported sperm recovery and delivery rates. The recent meta-analysis by Corona et al. estimated that eventually 16% of couples undergoing assisted reproduction for KS related azoospermia will have a child [10], yet the authors acknowledge that studies reporting retrieval rates and reproductive outcomes by intention-to-treat in single consecutive cohorts are lacking [10].

Therefore the present study aimed to analyse these outcomes in one large consecutive unselected cohort of KS patients undergoing a very first assisted reproduction treatment with TESE and ICSI.

## Materials and methods

### Patients

This retrospective cohort study analysed 138 KS patients having a first testicular biopsy between January 1994 and December 2013. KS patients with a previous history of TESE or with sperm in their ejaculate as well as mosaic KS were excluded. All patients had a clinical work-up with physical examination and endocrine profile (follicle stimulating hormone (FSH), luteinizing hormone (LH) and testosterone (T)). Scrotal ultrasound was performed in patients with a history of cryptorchidism. Patients on testosterone supplementation stopped their medication at least 6 months prior to any TESE procedure.

### Testicular sperm recovery

All patients had a testicular biopsy with multiple sampling under general anesthesia. The TESE procedure was performed either on the day of oocyte retrieval in the female partner (therapeutic), or prior to intervention in the female partner (diagnostic) combined with sperm cryopreservation when positive [11]. At our centre we work with standardized procedures both for testicular biopsy and processing of the testicular tissue samples in the laboratory. Two to five samples were taken from each testis depending on the testicular volume, either by random sampling or by micro-TESE procedure as described by Schlegel [12]. During surgery, one randomly taken biopsy of each testis was sent for histological analysis. The biopsies were collected in a Petri dish containing sperm buffer and delivered to the IVF laboratory. The testis biopsies were mechanically minced by using sterile microscope slides and/or needles in order to facilitate sperm release from the seminiferous tubules. Sperm search in this suspension was performed under the inverted microscope at 200x or 400x magnification. If no spermatozoa were observed after mechanical mincing, enzymatic digestion of the tissue was performed according to Crabbé et al. [13] in order to increase the chance to find sperm. The testicular cell suspension was frozen for later use if at least one spermatozoon was observed (diagnostic TESE) or if after injection of the mature oocytes at the day of oocyte retrieval supernumerary spermatozoa were still available for subsequent ICSI cycle(s) (therapeutic TESE) [11].

### Ovarian stimulation

In couples in whom testicular sperm was retrieved and frozen or in couples undergoing a combined TESE-ICSI procedure, female partners underwent ovarian stimulation using human menopausal or recombinant FSH in combination with GnRH (Gonadotrophin Releasing Hormone) agonist or antagonist according to standard protocols as described before [14].

### ICSI procedure

Fertilisation rates were expressed as the percentage of oocytes with two distinct pronuclei per injected metaphase II oocytes. Embryos were classified according to their morphological appearance. Normally cleaving embryos with less than 50% fragmentation were considered eligible for transfer. Up to 3 embryos were transferred into the uterine cavity at day 3, 4 or 5 after ICSI. From July 2003 onwards, the number of embryos transferred was restricted in accordance to Belgian Federal law on assisted reproductive technologies (ART), imposing single embryo transfer in women under 36 years of age [15]. Supernumerary embryos of sufficient quality were cryopreserved for later use.

Genetic counseling was offered to all couples and to patients opting for preimplantation genetic testing (PGT). PGT by fluorescence in situ hybridization (FISH) analysis on single blastomeres was carried out as previously described [16].

### Pregnancy follow-up

Pregnancy was diagnosed by elevated serum hCG levels (>15 IU/l) on at least two consecutive occasions. A clinical pregnancy was defined by the presence of a gestational sac at transvaginal ultrasound 5 weeks or later after embryo transfer. Patients with unknown outcome were considered not pregnant. The implantation rate was calculated as the number of gestational sacs with fetal heartbeat divided by the number of embryos transferred. A live birth was defined as a delivery of a live fetus after 20 completed weeks of gestational age or with a birth weight of ≥500g [17].

### Outcome measures

The primary outcome measure was the rate of deliveries resulting in live birth. The delivery of a singleton, twin or higher-order multiple pregnancies was registered as one delivery. Cumulative delivery rates were calculated, as this is the preferred measure reflecting the true effectiveness of assisted reproductive treatment [18]. Real (or observed) cumulative delivery rate is defined as the observed number of live birth deliveries after a pre-determined number of cycles, divided by the total number of couples who had ICSI treatment with testicular sperm. This outcome parameter is also called ‘crude cumulative delivery rate’. Only ICSI cycles reaching the stage of oocyte retrieval and with testicular sperm available for injection (fresh and/or frozen) were included in our analysis. A maximum of six treatment cycles per patient were considered. Patients with absence of testicular sperm at the moment of injection were not analyzed further. Moreover, all subsequent treatment cycles following the first live birth of a couple were excluded. When patients delivered following a transfer of supernumerary frozen embryos, this delivery was tallied up to the (unsuccessful) fresh ICSI cycle.

### Follow-up of pregnancy and children

Data on pregnancies, deliveries and neonatal histories were gathered form questionnaires filled out by parents and treating gynecologists. Children were invited for clinical assessment at the age of 3 months and 2 years to screen for congenital anomalies [17]. If performed, results of prenatal diagnosis or karyotyping of the children were collected.

### Statistical analysis

Baseline demographic patient characteristics were compared between men with positive and negative sperm retrieval. The Fisher exact test was used for categorical variables, while either the t-test (data presented with means ± standard deviation) or the Wilcoxon rank-sum test (data presented with medians and interquartile range) were used for all normally and non-normally distributed continuous variables, respectively. In order to assess which variables potentially affected the chances of a positive sperm retrieval, we performed stepwise forward logistic regression analysis accounting for male age and BMI and the following endocrine parameters: FSH, LH and T. A p<0.25 was the selection criteria for entering the final regression model.

Cycle characteristics and outcomes of non-PGT and PGT cycles were compared using logistic or linear regression adjusting standard errors for the clustering of cycles performed by the same couple. Fertilization and implantation rates were compared between these groups. However, given the limitations of simple linear and logistic regressions to evaluate such ratios [19], the mixed-effects multilevel regression analysis was performed, accounting for the two-level random effect potential for correlated outcomes from oocytes derived from the same ICSI cycle and also from cycles performed by the same couple. In order to minimize the potential effect of confounding variables on cumulative live birth rates, we also performed stepwise forward cox regression analysis accounting for female age, the number of COC retrieved, PGT and the year of treatment.

A p-value was considered significant if <0.05. For the statistical analysis, we used Stata Software version 13.1 (StataCorp, College Station, Texas, USA).

### Institutional review board approval

This retrospective study received approval by our Institute’s Ethics Committee. According to Belgian legislation, all data of patients were fully anonymised before analysis. Because the retrospective design of the study on anonymous patient data, no written informed consent of the patients was required according review by the local Ethical Committee.

## Results

### Testicular sperm recovery

**Fig 1:**
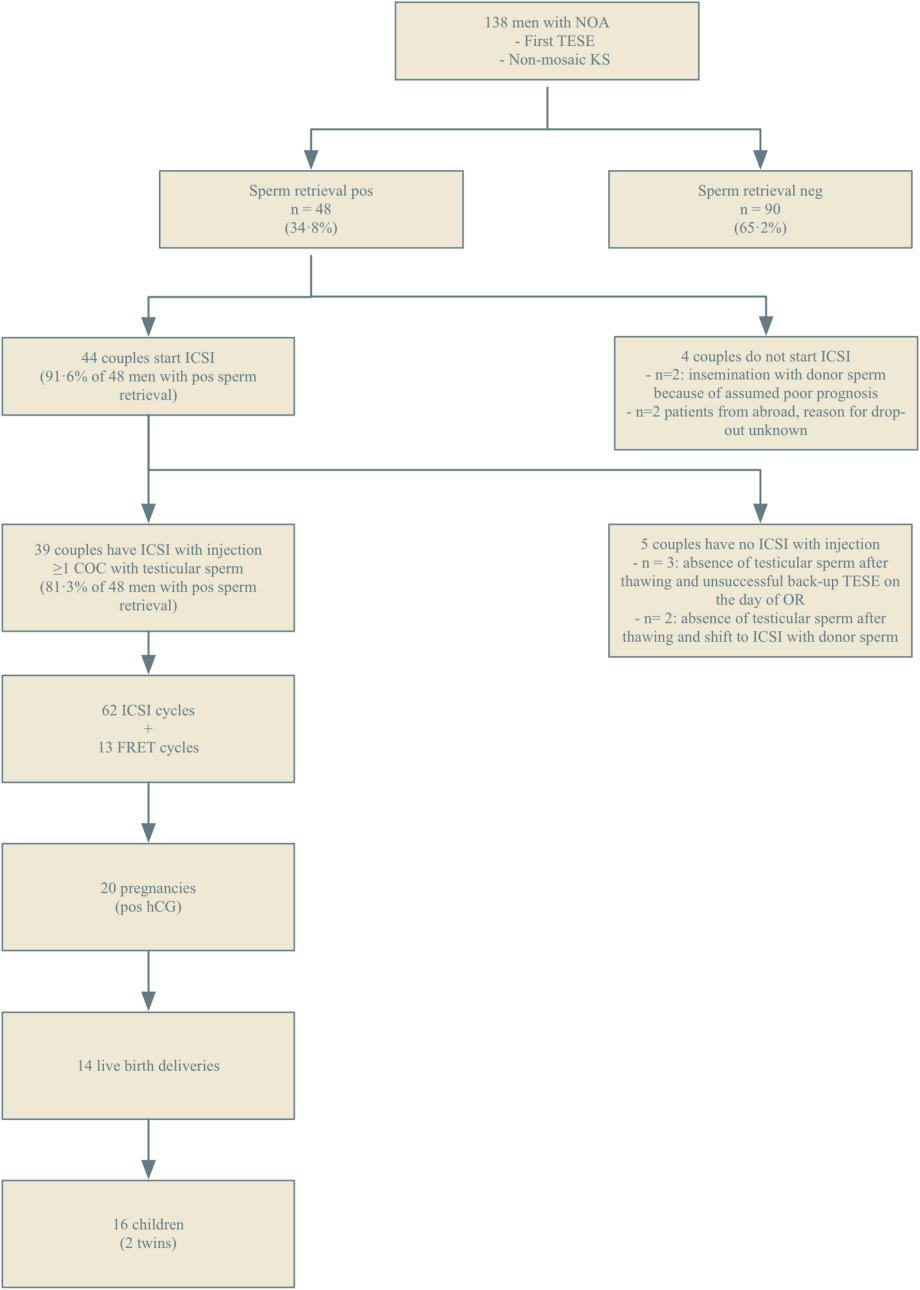
Schematic overview of the non-mosaic KS patients with azoospermia followed from first testicular sperm extraction until live birth delivery. NOA, non-obstructive azoospermia; KS, Klinefelter Syndrome; TESE, testicular sperm extraction; pos, positive; neg, negative; ICSI; intracytoplasmic sperm injection; COC, cumulus oocyte complex; OR, oocyte retrieval; FRET, frozen embryo transfer.

Out of the 138 unselected KS patients having their first testicular biopsy during the study period, 34.8% had testicular sperm retrieved (Fig 1). The demographic data, endocrine profile before TESE procedure, histopathological findings and planning of TESE procedures are presented in Table 1. Patients with a positive sperm retrieval were significantly younger (29 versus 33 years old, p<0.001), had a lower median BMI (23.8 versus 25.9, p = 0.002) and had significantly higher serum T measurements (9.7 versus 7.6 nmol/L, p = 0.001). However, following multivariable forward stepwise regression analysis (adjusted odds-ratio, 95% confidence interval), only male age (0.88, 0.80–0.97) and BMI (0.87, 0.79–0.97) remained significant predictors of sperm retrieval. The endocrine profile was not a significant predictor of sperm retrieval.

**Table 1:** Demographics, endocrine profile, histopathological findings and planning of TESE procedures in the 138 KS patients.

All patients (n = 138)	Pos. sperm retrieval	Neg. sperm retrieval	p-value
*Demographics* ^a^			
Age, yr	29.0 (26.5–33.0)	33.0 (30.0–37.0)	<0.001
BMI	23.8 (21.0–26.6)	25.9 (23.3–28.4)	0.002
*Endocrine profile* ^a^			
FSH (IU/L)	30.8 (22.1–37.5)	31.6 (22.8–40.6)	0.717
LH (IU/L)	16.6 (13.0–21.6)	18.6 (13.5–22.5)	0.525
T (nmol/L)	9.7 (7.7–12.5)	7.6 (5.2–11.1)	0.001
*TESE*			
**First TESE**	48 (34.8%)	90 (65.2%)	
Planning			0.28
Diagnostic	24 (50%)	54 (60%)	
Therapeutic	24 (50%)	36 (40%)	
Histopathology			0.075
SCO	11 (23%)	16 (18%)	
Sclerosis and atrophy	33 (69%)	69 (77%)	
MA	3 (6%)	0 (0%)	
Not done/representative	1 (2%)	5 (6%)	
**All TESE (1st + consecutive)**	62 (37.6%)	103 (62.4%)	
Time interval^b^	4.8 (3.5–8.7)	5.2 (4.2–14.5)	0.472
**Back-up TESE**^**c**^	5 (55.6%)	4 (44.4%)	

TESE, testicular sperm extraction; SCO, Sertoli cell-only; MA, maturation arrest; Pos., positive; Neg., negative; IQR, interquartile range; T, testosterone.

^a^ Data are presented as median (interquartile range).

^b^ Time interval means the interval between two TESE procedures; data are presented as mean (95% CI) months

^c^ New rescue TESE was performed if frozen-thawed suspensions could not be used on the day of oocyte retrieval.

### Outcome of ICSI with testicular sperm

Forty-four (44) couples started an ICSI trial either following a positive 'diagnostic' testicular biopsy with freezing, or in combination with a fresh testicular biopsy on the day of oocyte retrieval (therapeutic). Four couples in whom sperm was retrieved did not proceed to ICSI with the cryopreserved sperm: two opted for donor insemination while the other two (non-Belgian residents) never started ICSI for unknown reasons. Because in five couples no sperm for ICSI was available after thawing, eventually ICSI was performed in 39 couples with at least ≥1 mature oocyte injected with testicular sperm (Fig 1).

These 39 couples underwent 62 ICSI cycles and 13 associated frozen embryo transfer (FRET) cycles leading to 20 pregnancies (positive hCG, pregnancy rate: 32.3% per ICSI cycle with injection with testicular sperm– 29.9% per started ICSI cycle) and 14 live birth deliveries (live birth delivery rate: 22.6% per ICSI cycle with injection of testicular sperm– 20.9% per started ICSI cycle). The mean number of ICSI cycles performed per couple was 1.6. For those couples who delivered, the mean number of ICSI cycles needed to obtain a delivery was 1.3. Although follow-up of six cycles was planned, the maximum number of cycles performed per couple was four. Furthermore, live birth did not vary over time (p = 0.367).

Table 2 shows the treatment characteristics and ICSI outcome of the 62 ICSI cycles. Median female age was 27 years (Table 2). We performed a forward stepwise cox regression analysis using cumulative live birth as the outcome within the cycles performed, adjusting for female age, the number of oocytes retrieved, PGT and the year of treatment. None of these variables (including female age, hazard ratio 0.94, 95% confidence interval 0.82–1.08) remained in the final model as a significant predictor for cumulative live birth outcome.

**Table 2:** Treatment characteristics and ICSI outcome of patients with ≥ 1 oocyte injected with testicular sperm.

**All couples (n = 39)**				
Demographics				p-value
Age Female, yr ^a^	27 (26–31)			0.379
62 ICSI cycles				
Injection with fresh testicular sperm	32(51.6%)			
Injection with frozen testicular sperm	27(43.5%)			
Injection with testicular sperm (fresh and/or frozen) and donor sperm ^b^	3(4.8%)			
59 ICSI cycles (with only injection of testicular sperm)	all (n = 59)	non-PGT cycles (n = 29)	PGT cycles (n = 30)	p-value
***Treatment characteristics***				
Number of COCs ^c^	12.2 (±8.9)	10.5 (±7.0)	13.9 (±10.4)	0.111
Number of MII oocytes ^c^	9.1 (±5.2)	7.6 (±3.9)	10.5 (±7.0)	0.080
2PN (%)	311/533 (58.4)	110/202 (54.5)	201/331 (60.7)	0.339
Cycles with fertilization failure (n)	1	1	0	
Cycles with ET (n)	51	26	25	
Embryos (n)/ET ^c^	1.5(±0.6)	1.5(±0.6)	1.5(±0.6)	0.895
***Reproductive outcome*** ^d^				
Positive hCG/cycle (%)	18/59 (30.5)	10/29 (34.5)	8/30 (26.7)	0.515
Positive hCG/ET (%)	18/51 (35.3)	10/26 (38.5)	8/25 (32.0)	0.633
Clinical PR/cycle (%)	17/59 (28.8)	10/29 (34.5)	7/30 (23.3)	0.363
Clinical PR/ET (%)	17/51 (33.3)	10/26 (38.5)	7/25 (28.0)	0.455
Implantation rate (%)	16/77 (20.8)	10/39 (25.6)	6/38 (15.8)	0.343
Live birth delivery rate/cycle (%)	13/59 (22.0)	7/29 (24.1)	6/30 (20.0)	0.697
Live birth delivery rate/ET (%)	13/51 (25.5)	7/26 (26.9)	6/25 (24.0)	0.812

COC, cumulus oocyte complex; ET, embryo transfer; PR, pregnancy rate; PGT, preimplantation genetic testing

^a^ As recorded on the day of the oocyte retrieval at the first ICS cycle, median (IQR) years

^b^ Injection was done partially with testicular sperm and partially with donor sperm–the cycle was included since there was injection of resulting oocytes with testicular sperm, but in the calculation of the cumulative delivery rate only live birth delivery of injection with testicular sperm was included

^c^ Data are presented as mean (±SD)

^d^ Only outcome of fresh embryo transfers is given

The total number of oocytes retrieved was 765 of which 569 were mature. In eight cycles a fresh embryo transfer was not performed: in five cycles due to insufficient embryo quality, in one cycle because no genetically normal embryo available was after diagnosis, in two cycles with freeze all because of the risk of ovarian hyperstimulation syndrome (OHSS). Eighty-two embryos were transferred in a fresh cycle and 44 embryos were frozen. Thirteen frozen embryos transfer cycles (after unsuccessful fresh ICSI cycle) were planned of which two were canceled because of insufficient embryo survival after thawing.

**Fig 2:**
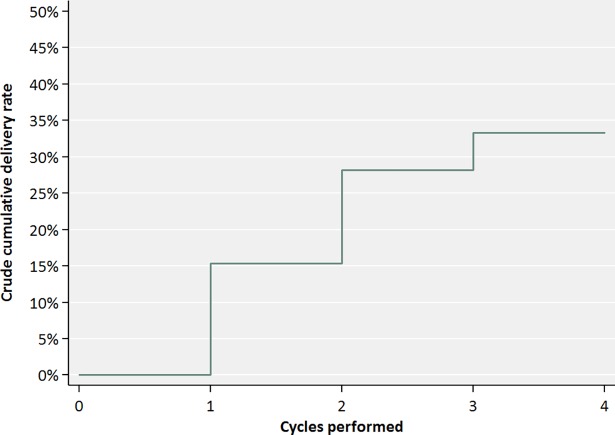
Crude cumulative delivery rates in non-mosaic azoospermic KS patients.

The crude cumulative delivery rate with a maximum of four ICSI cycles with testicular sperm was 35.9% (Fig 2 and Table 3). Although cumulative delivery rates are good once testicular sperm is available for ICSI, in this intention-to-treat analysis per patient, the actual chance per first TESE to have a live birth was limited to 10.1% (14/138). It is important to underline that in the latter analysis, patients who did not proceed to ICSI despite sperm being available at TESE, were also included.

**Table 3:** Crude cumulative delivery rates after ICSI with testicular sperm on non-mosaic azoospermic Klinefelter patients.

Treatment cycle number	1	2	3	4
Patients (n)	39	16	6	1
Non-delivery and discontinued (n)	16	4	3	1
Deliveries (n)	7	5	2	0
Non-delivery (n)	32	10	4	1
Drop-out rate	50%	40%	75%	100%
Crude cumulative delivery rate	18%	31%	36%	36%
Delivery rates per cycle	18%	31%	33%	0%

### Follow-up of pregnancy and children

Obstetric and neonatal outcome are shown in Table 4. Sixteen healthy children were born. Two patients were lost to follow-up and only written information was available of the delivery and health of the child at birth.

**Table 4:** Obstetric and neonatal outcome of the children born.

Treatment type	Delivered babies (n)	Term (w)	Weight (g)	Sex	Mode of delivery	Prenat/postnat karyotype
ICSI	2	38.1	2650, 2485	F, F	spontaneous	no/no
ICSI	1	41.0	4170	F	spontaneous	no/no
ICSI	1	40.4	3230	M	CS	no/46,XY
ICSI	1	40.5	3060	M	spontaneous	no/46,XY
ICSI	1	40.3	4140	M	spontaneous	no/no
ICSI	2	34.0	1900, 2250	F, F	CS	no/no
ICSI	1	38.5	?	M	CS	no/no
PGT	1	37.3	2800	M	CS	no/no
PGT	1	40.4	4300	F	spontaneous	no/46,XX
PGT	1	40.6	3390	F	vacuum	cvs/46,XX
PGT	1	39.5	3240	M	spontaneous	no/46,XY
PGT	1	41.2	3850	M	vacuum	no/46,XY
PGT	1	37.6	3650	F	spontaneous	no/no
FRET	1	35.4	2000	M	CS	no/no

CS, caesarean section; F, female; M, male; PGT, pre-implantation genetic testing; FRET, frozen embryo transfer; cvs, chorion villus sampling; prenat, prenatal; postnat, postnatal.

PGT was performed in six (42.9%) out of the 14 treatment cycles that resulted in a live birth. Chorion villus sampling was performed in one of the pregnancies and fetal karyotype was normal. Postnatal karyotyping was done in six children born and revealed a normal karyotype. No sex chromosomal abnormalities were found, but karyotypes were not available in all children born. One major malformation was reported in a girl diagnosed with retinitis pigmentosa. One minor malformation was reported in a girl, more specifically vesicoureteral reflux treated medically, not surgically. If surgery would prove to be necessary in the future, this malformation will need to be redefined as a major malformation (Table 4).

## Discussion

The current multidisciplinary follow-up of men diagnosed with KS focusses on long term quality of life and inevitably reproduction is part of this. Treating physicians will at some point be confronted with questions about reproductive performance. Hence, well-evidenced and unbiased information is important for adequate counseling. Since the introduction of TESE-ICSI in KS [9] these men are no longer labeled as infertile [20]. Although over the last two decades several reports have been published on sperm retrieval rates, only a few exist on live births after ICSI once testicular sperm were retrieved in the same cohort of patients. Furthermore, with the exception of a few studies, most are results on a selected patient population with live birth rates often higher than in patients with ejaculated sperm from oligozoospermic men, suggesting sampling bias. This is well illustrated in a recent meta-analysis: out of 23 studies, only five studies included more than 50 KS patients and nine reported a live birth rate per cycle exceeding 50% [10]. Therefore, counseling KS men on their realistic chances to become a biological father remains challenging, as figures available in the literature are prone to important publication and sampling bias and lack of comprehensive data [10].

The fertility treatment in KS men involves two major steps: firstly the surgical sperm retrieval and secondly the potential use of retrieved sperm for ICSI.

In literature, average sperm retrieval rate per TESE procedure is 44% with no differences between surgical methods of retrieval observed [10]. In our study the sperm retrieval rate was 34.8% per patient (sperm retrieval rate if only the first TESE is included) and 37.6% per procedure (sperm retrieval rate if all TESE procedures were included) (Table 1). These lower retrieval rates can be explained by the inclusion of unselected KS patients with no previous testicular biopsy in order to prevent selection bias. Indeed, prior testicular TESE with positive sperm retrieval or testicular histopathology showing mature spermatozoa is a known positive predictor for sperm retrieval in men with non-obstructive azoospermia (NOA) [21]. Mosaic KS patients were excluded as spermatogenesis in these cases has been reported as less affected [22]. Neither clinical nor hormonal parameters are known predictors of successful TESE [10]. A new finding of our study, as shown by regression analysis, is that BMI potentially interferes with sperm retrieval. The mechanism behind this could be an imbalance between oestradiol / testosterone ratio. Unfortunately, as oestradiol was not routinely assessed in our patients, future studies are needed to elucidate on this. In our dataset higher sperm retrieval rates were observed at younger age for the first TESE, with is in contrast with the data of Plotton et al. [23] and Corona et al. [10].

Considering step two of the TESE-ICSI approach, the ICSI procedure itself, clinical pregnancy rate and live birth rate per ICSI cycle were respectively 28.8% and 22% (Table 2). Sixteen healthy children were born, the karyotype being normal in 6 tested pre- or postnatally (Table 4). Of the 138 KS patients included 14 eventually fathered a genetically-own child, which amounts to 10.1%. These results are comparable with a previous study on 444 ICSI cycles at our centre in NOA patients having a normal karyotype showing a clinical pregnancy rate and live birth per cycle of respectively 21.7% and 20.6% and a cumulative delivery rate per patient embarking for TESE of 13.4% [24]. These figures are lower than those reported for KS patients in the literature: in the meta-analysis by Corona et al. an average live birth per cycle of 43% and an estimated cumulative delivery rate of 16% by intention-to-treat was reported in TESE positive couples undergoing ICSI [10]. As mentioned above, publication bias may account for this difference.

Very few studies report on large consecutive unselected series in their analysis. This study included a longitudinal cohort 138 KS patients analysed per intention-to-treat. The only comparable study is by Sabbaghian et al. including 134 patients reporting a sperm retrieval rate of 28.4% per KS patient (38/134) resulting in 4 deliveries from 18 ICSI cycles, or a cumulative delivery rate of 3% per patient [25]. Similarly, our patient population was unselected hence was not confined to good prognosis patients such as previous positive sperm retrieval or young female patients.

Bakircioglu et al. analysed 106 KS patients and reported a sperm retrieval rate of 47.2% (50/106), a live birth rate per cycle of 46.9% (23/49) and a cumulative delivery rate of 21.7% per patient [26]. However, in this study a selection towards female partners with good reproductive performance was made, which was not the case in our study.

Genetic counselling of couples with KS males remains a very important issue and in case of pregnancy after TESE-ICSI, prenatal diagnosis or a non-invasive prenatal test (NIPT) [27] should be discussed. Although NIPT is not yet validated for detection of sex-chromosome aneuploidies in all centres this technique becomes progressively more available to patients. The benefit of PGT in couples with KS males is debatable: there is no indication that embryos derived from KS patients show a higher prevalence of sex-chromosome aneuploidy [16]. Furthermore, our results in ICSI cycles comparing non-PGT cycles with PGT cycles did not show significant differences in implantation, clinical pregnancy and live birth rate per cycle (Table 2). Although in the past PGT practice may have reduced the number of precious embryos created with the few testicular sperm available in KS males, in our study no difference was seen in the number of cycles with embryos available for transfer between non-PGT and PGT cycles performed (Table 2).

In Belgium, TESE and up to 6 ICSI cycles are reimbursed with compulsory single embryo transfer in women less than 36 years. The (crude) cumulative delivery rate per couple having ICSI was 35.9% after four cycles (Table 2). Nevertheless, very few patients continued their treatment after the third and none after the fourth ICSI cycle. This illustrates that a combined TESE-ICSI approach imposes an important burden on both male and female partner in couples suffering from KS associated azoospermia.

Differences between our results and data found in literature can be explained by a different selection of patients (sampling bias), reallocation of patients with testicular sperm, smaller sample sizes and the cross sectional versus longitudinal study design in other studies. On the other hand, some limitations of our study should also be taken into account. Although being one of the largest studies currently available in the literature, the sample size remains small to allow conclusions towards predictive factors for sperm retrieval such as hormonal profile, age and BMI. Secondly, it is a retrospective analysis with a study period of 19 years. This study design was elected over such a long period as it was the only means to include enough patients into study of the cumulative live birth delivery rate after TESE-ICSI in a larger set of azoospermic non-mosaic KS males. Secular trends may therefore be present, however it is worthwhile mentioning that success rates did not change over time. Thirdly, technical improvements in laboratory techniques may be an important issue because in the past, live birth rates after transferring cryopreserved embryos by slow-freezing and thawing were substantially inferior to current vitrification results. However, few supernumerary embryos were available in our patients because of the limited number of spermatozoa available and the lower fertilisation rates. Furthermore, there is the limitation on the number of embryos to be transferred as legally imposed and single-embryo transfer may have lowered the cumulative delivery rate in younger patients using less efficient slow-freezing methods. A final limitation of the study is inherent to its observational character with information on the cause of patient dropouts not being available. However, one may argue that many of patients who eventually dropped out decided to do so because of the limited availability of sperm for ICSI.

KS requires a multidisciplinary approach in order to ensure a better quality of life (QOL). The developments in molecular genetics will allow, in the near future, more KS patients to be diagnosed [28]. For many KS reproductive performance is an important issue and many of them will enquire about this. Fertility counselling is an important part of the supportive processes in KS patients as reproduction is an inevitable part of QOL. Although TESE-ICSI is a major step in the treatment of infertility in non-mosaic KS men, the best cumulative delivery rates reported in the literature are not exceeding 25% per patient. In the present study, being one of the largest longitudinal follow-up studies reporting on unselected TESE in KS patients, this cumulative delivery rate figure is even lower.

Evidenced based predictive factors allowing selection of KS patients with a better profile are still lacking. In the meantime, prior to undergoing TESE for sperm recovery, KS patients should be counseled that eventually at least eight out of ten will probably not father a genetically-proper child when no selection criteria are applied at intake for TESE or eventual intake for ICSI.
